# The association between gallstones and metabolic syndrome in urban Han Chinese: a longitudinal cohort study

**DOI:** 10.1038/srep29937

**Published:** 2016-07-22

**Authors:** Qian Zhu, Xiubin Sun, Xiaokang Ji, Lin Zhu, Jing Xu, Chunxia Wang, Chengqi Zhang, Fuzhong Xue, Yanxun Liu

**Affiliations:** 1 Department of Epidemiology and Biostatistics, School of Public Health, Shandong University, Jinan 250012, Shandong, China; 2 The affiliated Hospital of Jining Medical University, Jining 272000, Shandong, China; 3 Shandong Provincial QianFoShan Hospital, Jinan 250012, Shandong, China

## Abstract

The precise association between metabolic syndrome (MetS) and gallstone disease remains unclear in China. This study aimed to clarify the relationship between MetS and gallstone and evaluate whether counts of metabolic abnormalities had influence on gallstone disease. We fitted gender-specific generalized estimating equation (GEE) regression models with data from a large-scale longitudinal study over 6-year follow-up to elucidate the real association. This study included 18291 participants with 3 times repeated measures at least who were free from a prior history of gallstone disease and cholecystectomy. A total of 873 cases of gallstones occurred during 6-year follow-up. The incidence density of gallstone in the group of subjects with MetS was higher than the group without MetS (10.27 vs 5.79). The GEE analyses confirmed and clarified the association between MetS and gallstone disease in males (RR = 1.33, P = 0.0020), while this association was not significant in females (RR = 1.15, P = 0.4962). With numbers of metabolic syndrome components increasing, the risk of gallstone disease showed corresponding increasing in males. In conclusion, the associations of MetS and gallstone are different in males and in females. And the risk of gallstone disease increases with the number of components of MetS for males but not for females.

With the economic development and the improvement of living standards in China in recent years, the prevalence of gallstone disease has been increasing gradually^1^. Gallstone disease is a common benign disease of biliary system, and most are asymptomatic. Nevertheless, it is associated with the potential risks of cholecystitis, pancreatitis, biliary tract obstruction and gall bladder cancer^2^,^3^. These complications contribute substantially to health care costs and can even threaten people’s life^4^. As a result of genetic and environmental factors, the prevalence of gallstone disease and the formation of gallstones are various throughout the world, due to the differences in region and race^5^,^6^,^7^. The high prevalence of gallstones not only increases the risk of complications and affects the quality of life^8^,^9^, but also consumes lots of economic and medical resources for society. For persons older than 20 years of age, the prevalence of gallstone disease is as high as 3.8% in southern China and 6.1% in northern China^10^.

Metabolic syndrome (MetS) is a group of metabolic diseases associated with insulin resistance, which is one of the major diseases that have a serious influence on human health. MetS refers to a combination of four basic medical disorders, including central obesity, raised fasting plasma glucose or post-prandial glucose, dyslipidemia and hypertension. With the change of living style of Chinese, the prevalence of MetS in China is rapidly increasing. According to a population-based cross-sectional survey in China in 2009, the crude and age-standardized prevalence of MetS was 31.5% and 30.5%, respectively^11^. Lifestyle modification and drug therapy can delay the development of MetS and prevalence of its components^12^,^13^.

For the last few years, many researches about the association between MetS and gallstones have been carried out around the world. Some researches described the risk factors about gallstones and MetS respectively^14^,^15^ and we discovered that they shared some common key risk factors, such as age, gender, overweight and disorders of lipid and glucose metabolism. Most of these factors are also key components for the diagnosis of MetS^16^. A Mexican cross-sectional study involving 245 subjects about the association between gallstone disease and MetS suggested that MetS was a risk factor for gallstone disease^17^. Moreover another study by Nervi, F with a nested case-control study on 881 Chilean subjects, demonstrated that gallstone disease might be a component of MetS^18^. The same results were reported on non-diabetic population and the elderly agricultural and fishing population in two Taiwan studies using hospital-based cross-sectional designs^19^,^20^. Another retrospective cross-sectional study in Taiwan concluded that MetS was associated with gallstone disease and gender difference might have different priorities and strategies to the burden of gallstone events^21^. Although previous studies have shown a positive relationship in Western population or in Taiwan area, China, a few studies have examined the relationship of MetS and gallstone events in mainland China. Because of heterogeneity existence, we had designed this study to explore the veritable relationship between MetS and gallstone disease in Han Chinese population.

Many studies tried to clarify this association, but they might be carried out without sufficient evidence. Some studies were conducted with cross-sectional designs^17^,^19^,^20^. To the best of our knowledge, cross-sectional designs may not be sufficient to capture the direct links between MetS and gallstone disease. Restricted to the inherent limitation of cross-sectional designs, cross-sectional studies using originally collected data which includes confounding variables are unable to deduce the relationship between the putative cause and effect. Therefore, longitudinal studies, especially those with large sample sizes, may well be appropriate to observe the changing trend and long-term influence between variables. Unlike cross-sectional design, a longitudinal study involves long-term follow-up and repeated observations of the same group of variables. Longitudinal studies make observing changes more accurate which can discover the potential association and the long-term effect of certain disease.

In this large-scale longitudinal-cohort study, we aimed to shed light on the relationship between MetS and the development of gallstone events and evaluate whether count of metabolic abnormalities was associated with gallstones in target population.

## Results

Out of 18291 participants, 873 individuals had gallstones in during a 6 years follow up. Mean age of 873 participants whose gallstone events occurred was 47.34 ± 14.04 years old, while 48.43 ± 14.19 years old for males and 45.44 ± 13.57 years old for females. As shown in Table 1, the distributions of four components of MetS (obesity, hypertension, diabetes mellitus and dyslipidemia) had strong differences (P < 0.0001) between two different groups separated by MetS status. Supplementary Table S1 showed the distribution of body mass index (BMI), systolic blood pressure (SBP), diastolic blood pressure (DBP), alanine aminotransferase (ALT), gamma-glutamyl transferase (GGT), total serum protein (STP), serum albumin (ALB), serum globulins (GLO), urea nitrogen (BUN), creatinine (CREA), glucose (GLU), total cholesterol (CHOL), triglycerides (TG), high density lipoprotein (HDL-C), low density lipoprotein (LDL-C), mean corpuscular-hemoglobin concentration (MCHC), neutrophilic granulocyte percentage, eosinophil percentage, basophil percentage, blood platelet (PLT) and diet. These potential confounding factors were statistically significant (P < 0.0001) between MetS group and non- MetS group at baseline survey.

Table 2 indicated the event rates per 1000 person-years of gallstones in 6 year follow-up between MetS group and non- MetS group. Of the 2679 individuals who were with MetS at baseline, 209 (7.80%) developed gallstones during the follow-up years, with the incidence density of 10.27 per 1000 person-years. For the individuals without MetS at baseline, 664 of 15612 (4.25%) developed gallstones, with the incidence density decreasing to 5.79 per 1000 person-years. The event rate of gallstones in participants with MetS was 1.77 times to the rate in ones without MetS. When subgroup analysis was performed in males and females, the event rates of gallstones in the MetS group were 10.29 and 10.16 per 1000 person-year respectively, and 6.27 and 5.08 per 1000 person-year in the non-MetS group, leading to the event rates with MetS 1.64 times higher in males than without MetS and 2.0 times higher in females than without MetS. The increasing risk of women with MetS was higher than the risk of men.

**Figure 1:**
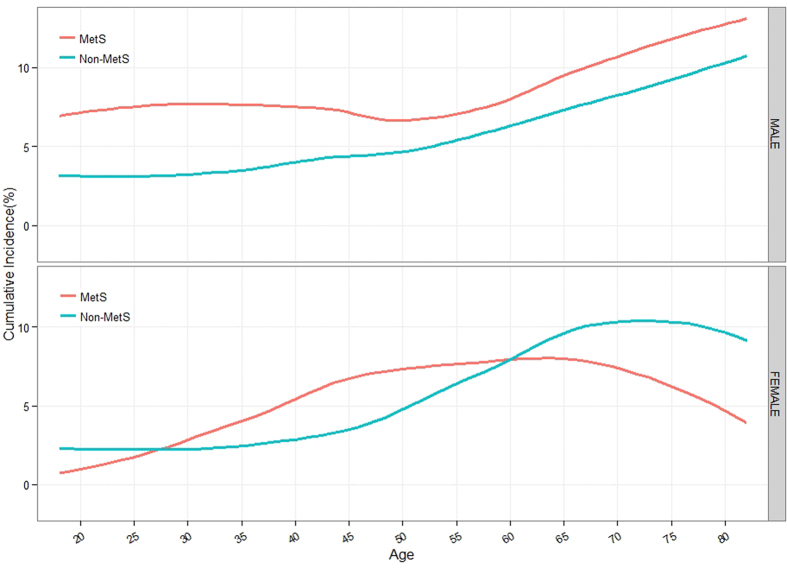
Modeled cumulative incidence of gallstone disease from age 18 to 82, stratified by sex and MetS status. The curves were modeled by real cumulative incidence of gallstone disease. Curves of cumulative incidence of gallstone disease among persons with MetS are in red; curves of cumulative incidence of gallstone disease among persons without MetS are in blue.

In Fig. 1, the gender-specific curves showed the cumulative incidence of gallstone from age 18 to 82 stratified by MetS status. For males, the cumulative incidence of gallstone had an increasing trend with age in group without MetS. While in group with MetS, it increased with age before 30 years, was stable between 30 and 40 years old, then decreased from 40 to 50 years old, and finally increased. The cumulative incidence of gallstone in group with MetS was higher than group without MetS in all age groups. For females, the cumulative incidence of gallstone increased with age from 35 years old and decreased at 75 years old in group without MetS. In group with MetS, it had an increasing trend with age before 50 years, was stable between 50 years old and 65 years old, and gradually decreased after 65 years old. The incidence of gallstone in group with MetS was higher than group without MetS before the age of 59 years old. On the contrary, the incidence of gallstone in group with MetS was lower than group without MetS after the age of 59 years old.

In the analysis of simple GEE models of gallstone disease, MetS had a statistically significant association (RR = 1.30, P = 0.0011) with gallstone disease for all participants after adjusting baseline age. Except dyslipidemia, the results of other metabolic components were in accordance with the result of MetS. Onset age of gallstone disease, BMI, blood pressure, ALT, GGT, STP, ALB, GLO, BUN, CREA, GLU, CHOL, LDL-C, MCHC, LYM, neutrophilic granulocyte percentage, basophilic granulocyte and diet were statistically significant in all participants. The results of simple GEE analysis for gallstone disease and MetS were showed in Supplementary Table S2. In gender-subgroup analyses (Supplementary Tables S3 and S4), MetS also had a significant association (RR = 1.33, P = 0.0020) with gallstone disease in males, but no significant association (RR = 1.15, P = 0.4962) between them in females. For metabolic components in males, the conclusions were in line with results in all participants. Whereas in females, only hypertension and diabetes mellitus had statistical association with gallstone event. The variables which were statistically significant were similar to all participants except for GGT, BUN, CHOL, LDL-C, lymphocyte (LYM), neutrophilic granulocyte percentage in males, and BMI, ALT, CREA, LDL-C, LYM, neutrophilic granulocyte percentage in females. MetS was associated with gallstone events (P = 0.0097) adjusted for potential confounding factors using multiple GEE model in all participants (Supplementary Table S5), and the significant association (P = 0.0011) also presented between MetS and gallstone disease in males (Supplementary Table S6). But in females, there was no statistical evidence to indicate positive relationship between them (Supplementary Table S7). Table 3 showed the results of age-adjusted simple GEE models and multiple GEE model adjusted for other potential confounding factors.

As shown in Table 4, MetS contained four metabolic disorders’ factors: overweight or obesity, high blood pressure, high blood sugar, blood lipid disorders. Participants were divided into 5 levels according to the counts of their metabolic disorders. Compared with Metabolic disorders’ 0 level- without any of four factors, RR values of 1, 2, 3, 4 levels were 1.26, 1.47, 1.50 and 2.60 respectively. Along with increased metabolic disorders’ levels, risk of gallstone disease also rose correspondingly, which showed that metabolic disorders had clear association with gallstone disease. In gender-subgroup analyses, after adjusting potential confounding factors, MetS also had a significant association with gallstone disease (P = 0.0011) in males, and the risk of gallstone disease in males was also increasing with the metabolic disorders’ levels, which was similar to that in all participants. The RR values were waved with the larger number of metabolic disorders’ levels in females and the risk of gallstone disease had no statistically significant increasing association with the counts of metabolic disorders (p > 0.05).

## Discussion

Both MetS and gallstones are common diseases in Chinese urban population. The relationship between MetS and gallstone disease had received much attention recently, but few studies had reported in Chinese population. In addition, most studies using cross-sectional or case-control designs might not be sufficient to explain the real link between MetS and gallstones^17^,^21^,^22^. More appropriate study design and statistical method should be adopted to assess this association. We carried out this study using a large scale longitudinal-cohort design in Chinese Han urban population. We found the incidence density of gallstone event in the group with MetS was higher than the incidence density in the group without MetS. As compatible with previous studies in Asian area, a significant association (RR = 1.25, P = 0.0097) was observed between MetS and gallstones development for all subjects in this 6 years follow-up study. The result can provide much guidance for the early warning and intervention strategies of gallstone event in patients with MetS in China.

We carried out a gender-specific study to research the relationship between MetS and gallstone. In this study, we found that the relationship was different in males and females. MetS was significantly associated with gallstone in all participants. The same relationship between MetS and gallstone was observed in males, but there was no statistical evidence about this association in females. Gallstone was a female-predominant disease^23^, while females had a lower risk for incident MetS than males^24^. This may explain why the relationship between MetS and gallstone disease in females was different from the relationship in males. According to the cumulative incidence curve of gallstone grouped by the status with or without MetS, age contributed to the development of gallstone and MetS was a risk factor for incidence of gallstone in males, which was consistent with some previous studies^22^. In females, the cumulative incidence had an increasing trend with age of up to 75 years old in participants without MetS, especially after 40 years old. The same conclusion was reported in the study by Gottfried Novacek and the reason of this phenomenon may be due to the fact that gallstone information was a cumulative process and rarely dissolved^25^. However, in group of participant with MetS, the cumulative incidence of gallstone was similar to a parabola and had no obvious increasing trend with age from 45 years old to 70 years old. And the relationship between the incidence of gallstone in group with MetS and the incidence in group without MetS was reversed at the age of 59 years old. This fact revealed the relationship between MetS and gallstone was maybe differ from premenopausal to postmenopausal participants of females. Considering the lag effect of menopause, estrogen may play a role in the relationship between MetS and incidence of gallstone. Pregnancy and estrogen increased the chances for gallstone disease and were the risk factors of gallstone^26^,^27^,^28^. Meanwhile, estrogen play a protective role against MetS and was associated with decreased risk of MetS^29^,^30^. The result of no significant association between MetS and gallstone may be due to the contrary effects of estrogen on MetS and gallstone disease. Further in-deep studies are needed to confirm the relationship between gallstone and MetS in females.

All subjects were separated in 5 levels based on the numbers of the metabolic disorders in our study. A significant positive trend between numbers of the components of MetS and prevalence of gallstone for males was discovered. The subjects with one, two, three and four metabolic factors suffered from the increasing risk of gallstone disease. This trend conformed to the previous studies, which showed a dose-dependent effect of metabolic abnormalities on the risk of gallstones^17^. More counts of metabolic disorders might lead to the higher prevalence of gallstones. However in females, as same as the association between gallstone and MetS, the risk of gallstone disease was not obviously increased with the counts of metabolic disorders. The effect of sex hormone on gallstone and MetS and the effect of each components of MetS may explain why no significant association between gallstone and the counts of metabolic disorders for females. This finding suggested that occurrence of gallstone disease was likely to happen after the development of metabolic disorders^19^, which indicated that clinicians should promote health measures for the prevention or early detection of gallstone disease in male, especially with larger number of metabolic disorders.

Several previous researches showed that gender contributed to the development of gallstone^20^,^25^. Females were possibly easier to occur gallstones and with higher prevalence of gallstones than males in some Western studies^23^,^31^. Estrogen was considered to be an obvious factor for gender difference in the epidemiologic study of gallstone disease in Western studies^32^. Although risk of gallstone in women with MetS was higher than the risk in men in this cohort study, there was no statistical evidence for the association between gender and prevalence of gallstones. Some studies among East Asian gallstone patients also supported this result which had failed to identify a gender-related difference^19^,^21^,^33^. The potential reason of which the relationship between gender and gallstone was controversial was likely to be that the compositions of gallstones were different for in Asia and Western countries^32^,^34^. Due to the correlation between hormone of females and cholesterol metabolites, females were a risk factor for cholesterol stone. But in China, pigment stone and mixed stone were more common which related to hemolysis, infection, and liver disease^35^,^36^,^37^.

In this research, we discussed the correlation separately between four components of metabolic disorders and gallstone disease. The results were as follows:

Obesity is not only a symptom but also a disorder which is a major factor in MetS. We also discovered that obesity had significant relationship with the development of gallstones, supported by previous studies^22^,^38^. Due to the increase in hepatic secretion of cholesterol and causing the bile to be supersaturated leading to the gallstones formation, obesity became a major risk factor for developing gallstones^6^,^22^.

Diabetes mellitus is a common component of MetS. This study illustrated that, there was a strong association between diabetes mellitus with gallstone events which was consistence with previous studies^39^,^40^,^41^. A population-based cohort study with 8 years follow-up in Taiwan, China, described the influence of diabetes mellitus on gallstones development and observed an increased risk of symptomatic gallstones diseases in diabetic patients^42^. Insulin resistance linked the hyperglycemia and gallstone formation. Hepatic insulin resistance was sufficient to increase biliary cholesterol secretion and promote cholesterol gallstone formation^34^. Meanwhile, hyperglycemia might affect gastrointestinal function and reduce the gallbladder contraction through vagal-cholinergic inhibition in response to various stimuli in subjects^43^, and then causing the gallstones.

Hypertension is a chronic medical condition which involves the systolic and/or diastolic pressures to be elevated. In this observational study, there was a positive relationship with high blood pressure and developing gallstones which was concordant with a study implemented in Taiwan, China^21^. Insulin resistance might be an important link for high blood pressure and gallstones which promoted hypertension^44^ and increases the rate of biliary cholesterol secretion and could finally cause gallstone events. The definite mechanism on how hypertension affects the development of gallstones is unclear and needs a future study.

The level of TG and HDC-C are diagnostic indicator of dyslipidemia. Although TG was higher and HDL-C was lower with the statistical difference in participants with MetS as compared with participants without MetS at baseline, this study failed to illustrate evident correlation between TG, HDL-C and the prevalence of gallstones adjusted age, as well as the result between dyslipidemia and gallstones. The relationships between blood-lipoids factors and gallstones were complex. A Korean study also did not find any evidence that any components of dyslipidemia could be correlated with the formation of gallstone disease^32^. Another cross-sectional study in China indicated that there was no conclusive link in HDL-C and developing gallstones^22^. But some studies in Western countries supported that lower HDL-C was a risk factor for gallstone events because a low serum HDL-C concentration was likely to associate with insulin resistance which led to the gallstones formation^45^,^46^. The reported mechanism how TG influenced the gallstone formation might be that higher TG also led to decreased gallbladder contraction and diminished gallbladder motility based on the supersaturated bile^47^. Cholesterol is the main chemical constituent in cholesterol gallstones which is the commonest type in Western countries, whereas calcium bilirubinate is the main constituent in pigment gallstones. In Eastern Asians, pigment stones and mix stones are more common^35^,^48^. Maybe the difference of distribution of gallstones’ types worldwide lead to the different results about the correlation between dyslipidemia and gallstones. Further researches are needed to indicate the definite relationships between components of dyslipidemia and gallstone development in Eastern Asians.

Based on a large-scale longitudinal cohort design, this research reveals long-term influence from study population confirming that MetS is an independent risk factor for gallstone development. However, there were several limitations in this study. First, owing to the shortcoming of health check-up database, we lacked full information of family history and genotypes. Therefore, we could not evaluate the influence of genetic factors to MetS and gallstone events. In addition, we were unable to obtain the information of pregnancy, estrogen, oral contraceptive pills and hormone replacement therapy in female patients who were considered to be associated with gallstones. The contributions of these information leading to gallstones in females were not evaluated. Furthermore, the information of lifestyle was incomplete in our study due to taking the self-report by participants for health questionnaire. The influence by the contribution of lacked information was not assessed. Finally, the participants of this cohort were only the urban inhabitants of middle to upper socioeconomic strata in Jinan, Shandong province and the results may not suit the general population in other regions and other ethnicity. Future cohort researches are needed to validate our results and to facilitate public health prevention or intervention program to reduce the incidence of gallstones disease.

## Conclusions

The significant association is observed between MetS and gallstone events for males but not for females in urban Han Chinese according to our study. With numbers of metabolic syndrome components increasing, the risk of gallstone diseases also shows corresponding increase for males after adjusting baseline age.

## Materials and Methods

### Study sample

**Figure 2:**
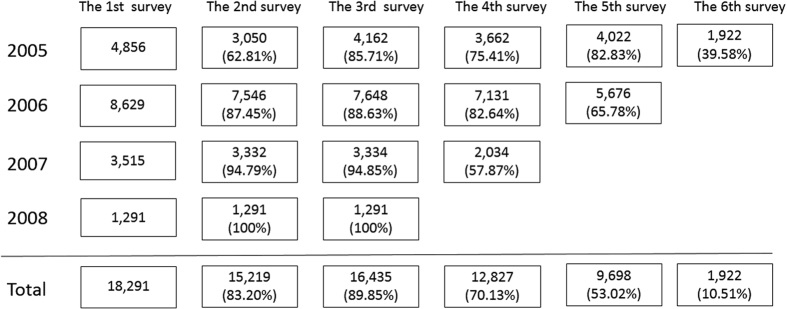
The distribution of participants for each repeated surveys at each year. The participants had their first survey and entered our cohort in 2005, 2006, 2007 and 2008, separately. The total number of participants was 18291. The 1st survey represented the total number of participants to be involved in the subsequent surveys of the different years. The 2^nd^ survey represented the number of participants who had their second survey in each subsequent year. Similarly, the 3^rd^ survey, the 4^th^ survey, the 5^th^ survey and 6^th^ survey separately represented the number of participants who had their third survey, fourth survey, fifth survey and sixth survey. 18291 participants took the 1st survey and at least two of 2^nd^–6^th^ surveys.

This prospective cohort study was based on the database from Centers for Health Management of Shandong Provincial Qianfoshan Hospital and Shandong Provincial Hospital which was the health information of participants who conducted the annual health examination in the center from 2005 to 2010. Participants of this cohort were from the middle-to-upper class urban citizens aged 18 to 82 years old in Jinan, the capital of Shandong province. A sub-cohort was selected from participants who took the first health check in 2005, 2006, 2007 or 2008 respectively and at least took health check-up 3 times in a 6-years follow-up. A total of 18291 participants (11611 men and 6680 women) who were free of gallbladder stone, biliary stone and cholecystectomy at their first health check-up were included in the sub cohort. Those participants were separated into two groups based on the status of metabolic syndrome at baseline and collected the information of gallstones and other factors in the follow-up years. Figure 2 showed the distribution of samples in our cohort for each repeated survey at each year and indicated the total of 18921 participants having at least three repeated health examination in the 6 years follow-up.

### Measurements

The health examination items included physical examination, laboratory tests, imaging tests, and health questionnaire. The physical examination contained weight, height, and blood pressure. The body mass index (BMI) was calculated as weight (kg) divided by squared height (m). All blood samples for laboratory tests were collected from participants who had fasted overnight at last 8 hours. Laboratory tests included blood tests, urine tests, stool tests and other special tests. Participants underwent the Doppler ultrasonography, computed tomography (CT), magnetic resonance imaging (MRI) or other imaging tests. Diet habits, smoking, alcohol intake and other variables were achieved from the health questionnaire. The diet habit was divided into 4 levels according to the mean food in daily life by retrospective self-report for participants. This study was approved by the Ethics Committee of School of Public Health, Shandong University, and conducted in accordance with the ethical guidelines of the Declaration of Helsinki of the World Medical Association. All participants gave written informed consents.

### Diagnostic criteria

According to the criteria given by the Chinese Medical Association Diabetes Branch (CDS) designed for Chinese^49^, MetS was defined as presence of three or more of the following four risk factors: 1) obesity/overweight, BMI ≥ 25.0 kg/m^2^; 2) hypertension, systolic/diastolic blood pressure ≥140/90 mmHg or previous diagnosis; 3) dyslipidemia, defined as fasting TG ≥ 1.7 mmol/L (110 mg/dl), or fasting high-density lipoprotein (HDL) < 0.9 mmol/L (35 mg/dl); 4) hyperglycemia, fasting blood-glucose (FPG) ≥ 6.1 mmol/L (110 mg/dl) or 2 h Post-meal glucose (PG) ≥ 7.8 mmol/L (140 mg/dl), or previous diagnosis.

Gallstone disease was confirmed by B-type ultrasonography. Gallstone was defined by the presence of strong intraluminal echoes that was gravity dependent or that attenuated ultrasound transmission (acoustic shadowing). Cholecystectomy was excluded from our study. Cholecystectomy was defined as the absence of the gallbladder on abdominal ultrasonography. We combined the surgical history of participants to decrease misdiagnosis rate of results by ultrasonography.

### Statistical analysis

#### Missing data imputation

Because of the lack of some physical examination or dropping out early of the participants, there were some variables with missing data. We used multiple imputations to account for missing values. According to the MI Procedure of SAS 9.4, the Markov chain Monte Carlo (MCMC) method was selected depending on the type of missing data and the imputed variables. Only data of covariates were imputed. After imputation, all covariates had less than 10% of missing proportion in particular, 1% for other variables except diet.

#### Data analysis

All statistical analyses were conducted by SAS version 9.4. Statistical descriptions were expressed as frequencies for variables at baseline status. Chi-square test was applied for variables to compare the differences between participants without MetS and participants with MetS at baseline. Firstly, baseline age-adjusted simple generalized estimating equation (GEE) models were used to select the interested factors associated with gallstones. Then, multiple GEE model was used to test the association between MetS and gallstones after adjusting potential confounders. Because MetS status was a binary dependent variable, ‘Logit’ was chosen as the link function for each GEE model. Furthermore, we studied the association between the amount of the four metabolic factors of MetS (i.e., obesity, hyperglycemia, hypertension and dyslipidemia) and gallstones. The relative risk (RR) with 95% CI was estimated simultaneously.

## Additional Information

**How to cite this article**: Zhu, Q. *et al*. The association between gallstones and metabolic syndrome in urban Han Chinese: a longitudinal cohort study. *Sci. Rep.* **6**, 29937; doi: 10.1038/srep29937 (2016).

## Supplementary Material

Supplementary Information

## Figures and Tables

**Table 1:** Distribution of associated factors and components of MetS for gallstone events of participants grouped by MetS status at baseline.

	non-Mets		Mets		Chiq	P value
	n	%	n	%		
Age at baseline					864.98	<0.0001
≤24	721	95.62	33	4.38		
25–44	9128	91.13	888	8.87		
45–64	4425	78.68	1199	21.32		
≥65	1338	70.53	559	29.47		
Gender					864.98	<0.0001
male	9365	80.66	2246	19.34		
female	6247	93.52	433	6.48		
Obesity					3148.59	<0.0001
No	9691	99.03	95	0.97		
Yes	5921	69.62	2584	30.38		
Hypertension					6005.73	<0.0001
No	13132	97.47	341	2.53		
Yes	2480	51.47	2338	48.53		
DM					3789.73	<0.0001
No	14891	90.83	1504	9.17		
Yes	721	38.03	1175	61.97		
Dyslipidemia					3135.99	<0.0001
No	10535	97.55	265	2.45		
Yes	5077	67.77	2414	32.23		

DM = diabetes mellitus.

**Table 2:** Event rates of gallstones in 6 year follow-up grouped by MetS status at baseline.

	MetS status	N (%)	Gallstone events%	Person-year observation	Event rate (per 1000 person-year)
Total	MetS	2679(14.65)	209(7.80)	20346	10.27
	non-MetS	15612(85.35)	664(4.25)	114626	5.79
Male	MetS	2246(19.36)	175(7.79)	17000	10.29
	non-MetS	9365(80.66)	429(4.58)	68385	6.27
Female	MetS	433(6.48)	34(7.85)	3346	10.16
	non-MetS	6247(93.52)	235(3.76)	46241	5.08

**Table 3:** Results of GEE analysis for gallstone disease and MetS.

	Age-adjusted				Multi-GEE			
	RR	lower 95% CI	higher 95% CI	P Value	RR	lower 95% CI	higher 95% CI	P Value
All	1.32	1.12	1.55	0.0011*	1.25	1.06	1.49	0.0097*
Male	1.33	1.11	1.59	0.0020*	1.36	1.13	1.63	0.0011*
Female	1.15	0.77	1.71	0.4962	0.84	0.56	1.26	0.4117

The association between MetS and gallstones was assessed by age-adjusted GEE analysis with the baseline age for all participants.

The association between MetS and gallstones was assessed by multiple GEE analysis with age, gender, ALT, GGT, STP, ALB, GLO, BUN, CREA, MCHC, LYM, neutrophil percentage, basophil percentage and diet history adjustment for all participants.

The association between MetS and gallstones was assessed by multiple GEE analysis with age, ALT, STP, ALB, GLO, CREA, MCHC, basophil percentage and diet history adjustment for male.

The association between MetS and gallstones was assessed by multiple GEE analysis with age, ALT, GGT, STP, ALB, GLO, BUN, MCHC, basophil percentage and diet history adjustment for female.

CI = Confidence interval; RR = the relative risk; *p < 0.05.

**Table 4:** The association between the count of metabolic disorders and gallstone events.

	Total				Male				Female			
	Number (yes/total)	Prevalence	RR (95% CI)	P value	Number (yes/total)	Prevalence	RR (95% CI)	P value	Number (yes/total)	Prevalence	RR (95% CI)	P value
Number of metabolic components				<0.0001				<0.0001				>0.05
0	174/5779	3.01	1		73/2352	3.1	1		101/3427	2.95	1	
1	229/5467	4.19	1.26 (1.04, 1.53)		155/3437	4.51	1.45 (1.12, 1.88)		74/2030	3.65	0.97 (0.72, 1.31)	
2	261/4366	5.93	1.47 (1.20, 1.79)		201/3576	5.62	1.5 (1.15, 1.95)		60/790	7.59	1.43 (0.97, 2.10)	
3	173/2205	7.85	1.5 (1.19, 1.89)		144/1858	7.75	1.62 (1.21, 2.16)		29/347	8.36	1.27 (0.78, 2.06)	
4	36/474	7.59	2.6 (1.94, 3.50)		31/388	7.99	2.87 (2.01, 4.09)		5/86	5.81	1.69 (0.84, 3.41)	

CI = Confidence interval; RR = the relative risk.
