# Supplementary material for: The association between gallstones and metabolic syndrome in urban Han Chinese: a longitudinal cohort study
Source: Sci Rep. 2016 Jul 22;6:29937. doi: 10.1038/srep29937 (PMC4957232; doi:10.1038/srep29937)
Supplement: Supplementary Information [file srep29937-s1.pdf]

The association between gallstones and metabolic syndrome in urban Han Chinese: a longitudinal cohort study

Qian Zhu[1], Xiubin Sun[1], Xiaokang Ji[1], Lin Zhu[1], Jing Xu[1], Chunxia Wang[2], Chengqi Zhang[3], Fuzhong Xue[1]*, Yanxun Liu[1]*

1 Department of Epidemiology and Biostatistics, School of Public Health, Shandong University, Jinan 250012, Shandong, China; E-Mails: zhu.qian@outlook.com (Z.Q.); sunxiubin@sdu.edu.cn (S.X.B); jxk@sdu.edu.cn (J.X.K); zhulin134@gmail.com (Z.L.); xujinghello@163.com (X.J.); xuefzh@sdu.edu.cn (X.F.Z.); liu-yx@sdu.edu.cn (L.Y.X.)

2 The affiliated Hospital of Jining Medical University, Jining 272000, Shandong, China; E-Mail: 414584600@qq.com (W.C.X.)

3 Shandong Provincial QianFoShan Hospital, Jinan 250012, Shandong, China；E-Mail: chengqizhangsd@126.com (Z.C.Q.)

*Corresponding author at:

Department of Epidemiology and Biostatistics, School of Public Health, Shandong University, 44 West Wenhua Road, Jinan 250012, Shandong, PR China

E-mail: liu-yx@sdu.edu.cn

Supplementary Table S1 Distribution of associated factors for gallstone events of participants grouped by MetS status at baseline

		non-Mets		Mets			
		n	%	n	%	Chiq	P value
BMI						3022.2	<.0001
	Normal	9560	98.93	103	1.07		
	High	6052	70.14	2576	29.86		
SBP						4218.80	<.0001

	Normal	13772	93.76	916	6.24		
	High	1840	51.07	1763	48.93		
DBP						2689.21	<.0001
	Normal	14879	89.7	1709	10.3		
	High	733	43.04	970	56.96		
ALT						288.13	<.0001
	Normal	14863	86.48	2324	13.52		
	Abnormal	749	67.84	355	32.16		
GGT						323.44	<.0001
	Normal	13990	87.11	2071	13.89		
	Abnormal	1622	72.74	608	27.26		
STP						14.92	0.0001
	Normal	14405	85.65	2413	14.35		
	Abnormal	1207	81.94	266	18.06		
ALB						31.53	<.0001
	Normal	15216	85.13	2658	14.87		
	Abnormal	396	94.96	21	5.04		
GLO						54.54	<.0001
	Normal	11457	86.55	1781	13.45		
	Abnormal	4155	82.23	898	17.77		
BUN						44.91	<.0001
	Normal	15354	85.14	2679	14.86		
	Abnormal	258	100	0	0		
CREA						49.85	<.0001
	Normal	13655	86.08	2209	13.92		
	Abnormal	1957	80.63	470	19.37		
GLU						2218.02	<.0001
	Normal	14145	90.42	1499	9.58		
	Abnormal	1467	55.42	1180	44.58		
CHOL						270.12	<.0001
	Normal	13353	87.25	1951	12.75		
	Abnormal	2259	75.63	728	24.37		
TG						2317.42	<.0001
	Normal	11187	94.67	630	5.33		
	Abnormal	4425	68.35	2049	31.65		
HDL-C						93.18	<.0001
	Normal	8680	87.68	1220	12.32		
	Abnormal	6932	82.61	1459	17.39		
LDL-C						235.05	<.0001
	Normal	11771	87.77	1640	12.23		
	Abnormal	3841	78.71	1039	21.29		
hemoglobin						0.93	0.3345
	Normal	14524	85.28	2506	14.72		
	Abnormal	1088	86.28	173	13.72		

MCHC						59.92	<.0001
	Normal	15103	85.01	2664	14.99		
	Abnormal	509	97.14	15	2.85		
WBC						2.13	0.1448
	Normal	14592	85.25	2524	14.75		
	Abnormal	1020	86.81	155	13.19		
LYM						0.46	0.5000
	Normal	14492	85.4	2477	14.6		
	Abnormal	1120	84.72	202	15.28		
neutrophil percentage						27.42	<.0001
	Normal	14986	85.08	2627	14.92		
	Abnormal	626	92.33	52	7.67		
eosinophil percentage						36.78	<.0001
	Normal	14774	84.99	2609	15.01		
	Abnormal	838	92.29	80	7.71		
basophil percentage						39.93	<.0001
	Normal	14995	85.03	2639	14.97		
	Abnormal	617	93.91	40	6.09		
PLT						42.39	<.0001
	Normal	13110	84.63	2381	15.37		
	Abnormal	2502	89.36	298	10.64		
diet						92.97	<.0001
	Vegetable-based	7093	87.64	1000	12.36		
	Balance	5478	82.19	1187	17.81		
	Meats-based	2999	86.23	479	13.77		
	Seafood	42	76.36	13	23.64		

BMI=body mass index; SBP=systolic blood pressure; DBP=diastolic blood pressure; ALT=alanine aminotransferase; GGT=gamma-glutamyl transferase; STP=total serum protein; ALB=serum albumin; GLO=serum globulins; BUN=urea nitrogen; CREA=creatinine; GLU=glucose; CHOL=total cholesterol; TG=triglycerides; HDL-C=high density lipoprotein; LDL-C=light density lipoprotein; MCMC=mean corpuscular-hemoglobin concentration; LYM=lymphocyte; WBC=white blood cell; PLT=blood platelet.

Supplementary Table S2 The results of simple GEE Models of all participants after adjusting age at baseline

Variable	Estimate	Standard Error	Z	P Value	RR (95% CI)
MetS	0.28	0.0844	3.27	0.0011*	1.32 (1.12, 1.55)
Obesity	0.23	0.0714	3.29	0.0010*	1.26 (1.10, 1.45)
hypertension	0.58	0.0820	7.03	<.0001**	1.78 (1.52, 2.09)
DM	0.46	0.0866	5.34	<.0001**	1.59 (1.34, 1.88)
dyslipidemia	-0.09	0.0672	-1.35	0.1762	0.91 (0.80, 1.04)
gender	-0.14	0.0774	-1.86	0.0628	0.87 (0.74, 1.01)
age	0.48	0.0176	27.32	<.0001**	1.62 (1.56, 1.67)
BMI	0.04	0.0104	4.11	<.0001**	1.04 (1.02, 1.07)

SBP	0.01	0.0018	6.32	<.0001**	1.01 (1.01, 1.01)
DBP	0.01	0.0032	2.79	0.0053*	1.01 (1.00, 1.02)
ALT	0.00	0.0004	2.67	0.0076*	1.00 (1.00, 1.00)
GGT	0.00	0.0005	2.72	0.0065*	1.00 (1.00, 1.00)
STP	0.03	0.0069	3.69	0.0002*	1.03 (1.01, 1.04)
ALB	-0.06	0.0120	-5.4	<.0001**	0.94 (0.92, 0.96)
GLO	0.05	0.0071	7.38	<.0001**	1.05 (1.04, 1.07)
BUN	0.07	0.0252	2.77	0.0056*	1.07 (1.02, 1.13)
CREA	0.00	0.0017	2.13	0.0334*	1.00 (1.00, 1.01)
GLU	0.11	0.0198	5.66	<.0001**	1.12 (1.08, 1.16)
CHOL	0.09	0.0329	2.79	0.0053*	1.10 (1.03, 1.17)
TG	0.04	0.0212	1.76	0.0776	1.04 (1.00, 1.08)
HDL-C	0.13	0.1050	1.21	0.2272	1.14 (0.92, 1.39)
LDL-C	0.12	0.0461	2.6	0.0093*	1.13 (1.03, 1.23)
hemoglobin	0.00	0.0024	0.85	0.3972	1.00 (1.00, 1.01)
MCHC	0.02	0.0030	6.07	<.0001**	1.02 (1.01, 1.02)
WBC	0.00	0.0210	-0.12	0.908	1.00 (0.96, 1.04)
LYM	0.11	0.0538	2.10	0.0357*	1.12 (1.01, 1.24)
neutrophil percentage	-0.01	0.0043	-2.26	0.0238*	0.99 (0.98, 1.00)
eosinophil percentage	0.03	0.0172	1.92	0.0551	1.03 (1.00, 1.07)
basophil percentage	-0.60	0.1653	-3.62	0.0003*	0.55 (0.40, 0.76)
PLT	0.00	0.0006	1.56	0.1179	1.00 (1.00, 1.00)
diet					
Vegetable-based	0.00	0	Ref	Ref	Ref
Balance	-0.07	0.1019	-0.70	0.4830	0.93 (0.76, 1.14)
Meats-based	0.39	0.0765	5.16	<.0001**	1.48 (1.28, 1.72)
Seafood	0.60	0.3675	1.63	0.1029	1.82 (0.89, 3.74)

DM= diabetes mellitus. BMI=body mass index; SBP=systolic blood pressure; DBP=diastolic blood pressure; ALT=alanine aminotransferase; GGT=gamma-glutamyl transferase; STP=total serum protein; ALB=serum albumin; GLO=serum globulins; BUN=urea nitrogen; CREA=creatinine; GLU=glucose; CHOL=total cholesterol; TG=triglycerides; HDL-C=high density lipoprotein; LDL-C=light density lipoprotein; MCMC=mean corpuscular-hemoglobin concentration; LYM=lymphocyte; WBC=white blood cell; PLT=blood platelet.

P values were adjusted for age at baseline; *$p < 0.05$, **$p < 0.0001$; CI = Confidence interval; RR=the relative risk.

Supplementary Table S3 The results of simple GEE Models of male after adjusting age at baseline

Variable	Estimate	Standard Error	Z	P Value	RR (95% CI)
MetS	0.28	0.0918	3.09	0.002*	1.33 (1.11, 1.59)
Obesity	0.24	0.0858	2.78	0.0054*	1.27 (1.07, 1.50)
hypertension	0.56	0.0937	5.96	<.0001**	1.75 (1.45, 2.10)
DM	0.45	0.0987	4.53	<.0001**	1.56 (1.29, 1.90)
dyslipidemia	-0.13	0.0788	-1.60	0.1091	0.88 (0.76, 1.03)
age	0.49	0.0219	22.46	<.0001**	1.64 (1.57, 1.71)
BMI	0.05	0.015	3.12	0.0018*	1.05 (1.02, 1.08)

SBP	0.01	0.0021	5.24	<.0001**	1.01 (1.01, 1.02)
DBP	0.01	0.0027	2.82	0.0047*	1.01 (1.00, 1.01)
ALT	0.00	0.0008	2.44	0.0145*	1.00 (1.00, 1.00)
GGT	0.00	0.0007	1.04	0.2986	1.00 (1.00, 1.00)
STP	0.02	0.0083	2.07	0.0388*	1.02 (1.00, 1.03)
ALB	-0.07	0.0149	-4.83	<.0001**	0.93 (0.90, 0.96)
GLO	0.05	0.0085	5.33	<.0001**	1.05 (1.03, 1.06)
BUN	0.05	0.0331	1.38	0.1684	1.05 (0.98, 1.12)
CREA	0.00	0.0018	2.06	0.0397*	1.00 (1.00, 1.01)
GLU	0.10	0.0247	4.08	<.0001**	1.11 (1.05, 1.16)
CHOL	0.05	0.0419	1.27	0.2039	1.05 (0.97, 1.14)
TG	0.02	0.0271	0.72	0.4719	1.02 (0.97, 1.08)
HDL-C	0.18	0.1327	1.35	0.1759	1.20 (0.92, 1.55)
LDL-C	0.05	0.0587	0.91	0.3647	1.05 (0.94, 1.18)
hemoglobin	0.00	0.0040	-0.62	0.5300	1.00 (0.99, 1.01)
MCHC	0.02	0.0040	4.47	<.0001**	1.02 (1.01, 1.03)
WBC	-0.02	0.0259	-0.59	0.5568	0.98 (0.94, 1.04)
LYM	0.09	0.0638	1.41	0.1591	1.09 (0.97, 1.24)
neutrophil percentage	-0.01	0.0052	-1.95	0.0509	0.99 (0.98, 1.00)
eosinophil percentage	0.03	0.0216	1.24	0.2155	1.03 (0.98, 1.07)
basophil percentage	-0.57	0.202	-2.83	0.0047*	0.56 (0.38, 0.84)
PLT	0.00	0.0008	0.45	0.6501	1.00 (1.00, 1.00)
diet					
Vegetable-based	0.00	0	Ref	Ref	Ref
Balance	-0.11	0.1188	-0.89	0.3727	0.90 (0.71, 1.14)
Meats-based	0.31	0.0963	3.27	0.0011*	1.37 (1.13, 1.65)
Seafood	0.58	0.4235	1.37	0.1701	1.79 (0.78, 4.10)

DM= diabetes mellitus. BMI=body mass index; SBP=systolic blood pressure; DBP=diastolic blood pressure; ALT=alanine aminotransferase; GGT=gamma-glutamyl transferase; STP=total serum protein; ALB=serum albumin; GLO=serum globulins; BUN=urea nitrogen; CREA=creatinine; GLU=glucose; CHOL=total cholesterol; TG=triglycerides; HDL-C=high density lipoprotein; LDL-C=light density lipoprotein; MCMC=mean corpuscular-hemoglobin concentration; LYM=lymphocyte; WBC=white blood cell; PLT=blood platelet.
P values were adjusted for age at baseline; *p < 0.05, **p < 0.0001; CI = Confidence interval; RR=the relative risk.

Supplementary Table S4 The results of simple GEE Models of female after adjusting age at baseline

Variable	Estimate	Standard Error	Z	P Value	RR (95% CI)
MetS	0.14	0.2037	0.68	0.4962	1.15 (0.77, 1.71)
Obesity	0.08	0.1495	0.54	0.5863	1.08 (0.81, 1.45)
hypertension	0.53	0.1584	3.37	0.0007*	1.71 (1.25, 2.33)
DM	0.47	0.1709	2.76	0.0058*	1.60 (1.15, 2.24)
dyslipidemia	-0.14	0.1364	-1.00	0.3179	0.87 (0.67, 1.14)
age	0.45	0.0285	15.82	<.0001**	1.57 (1.48, 1.66)

BMI	0.01	0.0213	0.70	0.4851	1.02 (0.97, 1.06)
SBP	0.01	0.0034	3.00	0.0027*	1.01 (1.00, 1.02)
DBP	0.01	0.0059	1.90	0.0572	1.01 (1.00, 1.02)
ALT	0.00	0.0004	1.67	0.0945	1.00 (1.00, 1.00)
GGT	0.00	0.0016	2.88	0.0039*	1.00 (1.00, 1.01)
STP	0.04	0.0131	3.07	0.0021*	1.04 (1.01, 1.07)
ALB	-0.07	0.021	-3.32	0.0009*	0.93 (0.90, 0.97)
GLO	0.07	0.0134	5.46	<.0001**	1.08 (1.05, 1.10)
BUN	0.09	0.0453	2.00	0.0454*	1.09 (1.00, 1.20)
CREA	0.00	0.0043	0.33	0.7445	1.00 (0.99, 1.01)
GLU	0.13	0.0342	3.78	0.0002*	1.14 (1.06, 1.22)
CHOL	0.14	0.0594	2.38	0.0172*	1.15 (1.03, 1.29)
TG	0.04	0.0424	0.96	0.3348	1.04 (0.96, 1.13)
HDL-C	0.20	0.1854	1.10	0.2700	1.23 (0.85, 1.76)
LDL-C	0.15	0.1041	1.43	0.1528	1.16 (0.95, 1.42)
hemoglobin	0.00	0.0049	-0.46	0.6428	1.00 (0.99, 1.01)
MCHC	0.02	0.0048	4.03	<.0001**	1.02 (1.01, 1.03)
WBC	0.00	0.0373	0.13	0.8989	1.00 (0.93, 1.08)
LYM	0.10	0.1088	0.88	0.3784	1.10 (0.89, 1.36)
neutrophil percentage	-0.01	0.008	-0.81	0.4157	0.99 (0.98, 1.01)
eosinophil percentage	0.05	0.0319	1.42	0.1544	1.05 (0.98, 1.11)
basophil percentage	-0.59	0.2976	-1.99	0.0466*	0.55 (0.31, 0.99)
PLT	0.00	0.0009	2.69	0.0071*	1.00 (1.00, 1.00)
diet					
Vegetable-based	0.00	0	Ref	Ref	Ref
Balance	-0.20	0.2252	-0.90	0.3679	0.82 (0.53, 1.27)
Meats-based	0.44	0.136	3.25	0.0012*	1.56 (1.19, 2.03)
Seafood	0.39	0.7155	0.54	0.5866	1.48 (0.36, 6.00)

DM= diabetes mellitus. BMI=body mass index; SBP=systolic blood pressure; DBP=diastolic blood pressure; ALT=alanine aminotransferase; GGT=gamma-glutamyl transferase; STP=total serum protein; ALB=serum albumin; GLO=serum globulins; BUN=urea nitrogen; CREA=creatinine; GLU=glucose; CHOL=total cholesterol; TG=triglycerides; HDL-C=high density lipoprotein; LDL-C=light density lipoprotein; MCMC=mean corpuscular-hemoglobin concentration; LYM=lymphocyte; WBC=white blood cell; PLT=blood platelet.

P values were adjusted for age at baseline; *p < 0.05, **p < 0.0001; CI = Confidence interval; RR=the relative risk.

Supplementary Table S5 The results of multiple GEE of all participants after adjusting confounding factors

Variable	Estimate	Standard Error	Z	P Value	RR (95% CI)
Intercept	-9.35	1.3832	-6.76	<.0001	
MetS	0.23	0.0871	2.58	0.0097	1.25 (1.06, 1.49)
age	0.03	0.0027	9.82	<.0001	1.03 (1.02, 1.03)

gender	0.03	0.0942	0.31	0.7599	1.03 (0.86, 1.24)
ALT	0.00	0.0004	1.77	0.076	1.00 (1.00, 1.00)
GGT	0.00	0.0006	1.21	0.2257	1.00 (1.00, 1.00)
STP	-0.12	0.067	-1.77	0.0772	0.89 (0.78, 1.01)
ALB	0.09	0.0681	1.38	0.1661	1.10 (0.96, 1.26)
GLO	0.16	0.0666	2.34	0.0195	1.17 (1.03, 1.33)
BUN	0.05	0.0307	1.59	0.1113	1.05 (0.99, 1.12)
CREA	0.00	0.0023	1.05	0.2957	1.00 (1.00, 1.01)
MCHC	0.01	0.0033	3.84	0.0001	1.01 (1.01, 1.02)
LYM	-0.05	0.0704	-0.65	0.5179	0.96 (0.83, 1.10)
neutrophil percentage	-0.01	0.0055	-2.72	0.0064	0.99 (0.97, 1.00)
basophil percentage	-0.79	0.1767	-4.49	<.0001	0.45 (0.32, 0.64)
diet					
Seafood	0.60	0.4136	1.45	0.1461	1.82 (0.81, 4.10)
Meats-based	0.38	0.0839	4.52	<.0001	1.46 (1.24, 1.72)
Balance	-0.09	0.1081	-0.80	0.4221	0.92 (0.74, 1.13)
Vegetable-based	0	0	Ref	Ref	Ref

ALT=alanine aminotransferase; GGT=gamma-glutamyl transferase; STP=total serum protein; ALB=serum albumin; GLO=serum globulins; BUN=urea nitrogen; CREA=creatinine; MCMC=mean corpuscular-hemoglobin concentration; LYM=lymphocyte.

P values were adjusted for other potential confounding factors; CI = Confidence interval; RR=the relative risk.

Supplementary Table S6 The results of multiple GEE of male after adjusting confounding factors

Variable	Estimate	Standard Error	Z	P Value	RR (95% CI)
Intercept	-9.48	1.6662	-5.69	<.0001	
MetS	0.31	0.0939	3.26	0.0011	1.36 (1.13, 1.63)
age	0.02	0.0031	7.41	<.0001	1.02 (1.02, 1.03)
ALT	0.00	0.0011	1.23	0.2177	1.00 (1.00, 1.00)
STP	1.73	1.3883	1.25	0.2124	5.65 (0.37, 85.82)
ALB	-1.75	1.3893	-1.26	0.2077	0.17 (0.01, 2.64)
GLO	-1.70	1.3887	-1.22	0.2211	0.18 (0.01, 2.78)
CREA	0.00	0.0022	1.37	0.1711	1.00 (1.00, 1.01)
MCHC	0.01	0.0041	2.72	0.0065	1.01 (1.00, 1.02)
basophil percentage	-0.75	0.2105	-3.56	0.0004	0.47 (0.31, 0.71)
diet					
Seafood	0.74	0.4451	1.66	0.097	2.09 (0.87, 5.01)
Meats-based	0.37	0.1043	3.51	0.0004	1.44 (1.18, 1.77)
Balance	-0.08	0.1241	-0.63	0.5279	0.92 (0.73, 1.18)
Vegetable-based	0	0	Ref	Ref	Ref

ALT=alanine aminotransferase; STP=total serum protein; ALB=serum albumin; GLO=serum globulins; CREA=creatinine; MCMC=mean corpuscular-hemoglobin concentration.

P values were adjusted for other potential confounding factors; CI = Confidence interval; RR=the relative risk.

Supplementary Table S7 The results of multiple GEE of female after adjusting confounding factors

Variable	Estimate	Standard Error	Z	P Value	RR (95% CI)
Intercept	-9.98	2.174	-4.59	<.0001	
MetS	-0.17	0.2063	-0.82	0.4117	0.84 (0.56, 1.26)
age	0.03	0.005	6.94	<.0001	1.04 (1.03, 1.05)
ALT	0.00	0.0012	-0.03	0.9786	1.00 (1.00, 1.00)
GGT	0.00	0.0017	2.4	0.0166	1.00 (1.00, 1.01)
STP	-0.16	0.0551	-2.9	0.0038	0.85 (0.77, 0.95)
ALB	0.12	0.0589	1.95	0.0508	1.12 (1.00, 1.26)
GLO	0.21	0.0528	4.06	<.0001	1.24 (1.12, 1.37)
BUN	0.08	0.0409	1.93	0.0535	1.08 (1.00, 1.17)
MCHC	0.01	0.0055	2.09	0.0366	1.01 (1.00, 1.02)
basophil percentage	-0.52	0.3025	-1.73	0.083	0.59 (0.33, 1.07)
Diet					
Seafood	0.04	1.1725	0.03	0.9730	1.04 (0.10, 10.36)
Meats-based	0.39	0.1411	2.73	0.0064	1.47 (1.11, 1.94)
Balance	-0.23	0.2317	-1.01	0.3128	0.79 (0.50, 1.25)
Vegetable-based	0	0	Ref	Ref	Ref

ALT=alanine aminotransferase; GGT=gamma-glutamyl transferase; STP=total serum protein; ALB=serum albumin; GLO=serum globulins; BUN=urea nitrogen; MCMC=mean corpuscular-hemoglobin concentration.

P values were adjusted for other potential confounding factors; CI = Confidence interval; RR=the relative risk.
